# Effects on gait kinematics, pedobarography, functional and subjective results after isolated chopart injury

**DOI:** 10.1186/s12891-024-07467-1

**Published:** 2024-04-26

**Authors:** Charlotte Cibura, Raimund Lülsdorff, Thomas Rosteius, Alexis Brinkemper, Maria Bernstorff, Birger Jettkant, Periklis Godolias, Tim Ramczykowski, Matthias Königshausen, Thomas A. Schildhauer, Christiane Kruppa

**Affiliations:** Department of General and Trauma Surgery, BG-University Hospital Bergmannsheil, Ruhr-University, Bochum, Germany Bürkle-de-la-Camp Platz 1, 44789

**Keywords:** Chopart injury, Gait analysis, Pressure distribution, Midfoot, Fracture

## Abstract

**Background:**

This study analysed changes in gait and pedobarography and subjective and functional outcomes after isolated Chopart joint injury.

**Methods:**

The results of 14 patients were reviewed. Kinematic 3D gait analysis, comparative bilateral electromyography (EMG) and pedobarography were performed.

**Results:**

On the injured side, the 3D gait analysis showed a significantly increased internal rotation and decreased external rotation of the hip and significantly decreased adduction and decreased range of motion (ROM) for the ankle. On the healthy side, the pedobarography revealed a significantly increased mean force in the forefoot, an increased peak maximum force and an increased maximum pressure in the metatarsal. When standing, significantly more weight was placed on the healthy side.

The EMG measurements showed no significant differences between the healthy and injured legs.

**Conclusions:**

After isolated Chopart injuries, significant changes in gait and pedobarography can be seen over the long term.

## Background

Injuries of the Chopart joint are rare at 2.2 to 3.6/100000/year, and they are overlooked and/or misinterpreted in up to 40% of cases despite advanced diagnostic possibilities [1–6]. The cause of Chopart injuries is either high-energy trauma, such as traffic accidents/falls from heights (more common in younger men), or low-energy trauma, such as sprain or sports injuries (more common in women) [2, 5]. In the case of displaced fractures or dislocations, open reduction and fixation with restoration of the joint surface and the medial and lateral foot column is recommended [7–12]. Nondisplaced fractures or avulsions are usually subject to conservative therapy. However, even apparently harmless avulsions can indicate complex injuries to the entire series of joints and can lead to further complications such as arthrodesis, which is why the injuries should not be underestimated [13–15]. For the physiological gait, however, the Chopart joint has an important function, especially in the stance phase of the gait cycle when the foot supports body weight. Furthermore, it optimizes adaptation to uneven ground during the first heel contact and stabilizes the foot at the end of the gait cycle for a firm push-off without loss of power [7, 9, 16]. The extent to which isolated injuries of the Chopart joint influence the entire gait pattern of the lower extremities and thus possibly lead to other consequences has not been adequately investigated to date. The aim of the study was therefore to investigate whether any type of injury in the Chopart series has an impact on the overall gait pattern and the joints of the lower extremities.

## Methods

The present study was performed in accordance with the Declaration of Helsinki and its later amendments. Ethical permission for this study was obtained from the ethics committee (registration number: 20–6865-§ 23b).

### Patients

Patients with an unilateral injury to the Chopart Joint treated in our hospital (level 1 trauma centre) from 01/2008 until 12/2019 were eligible, more precisely, all patients with a fracture or bony avulsion at the anterior process of the calcaneus (APC), cuboid, talar head, and/or navicular bone. The classification for Chopart injuries according to Main and Jowett was used as a guide, which states that these injuries vary from small avulsion fragments to severe sub/luxations [12]. Patients with injuries that were initially missed and delayed treated were also included. The exclusion criteria were as follows:

1. Additional fractures outside the Chopart joint at both feet
2. Other fractures, joint prostheses or other gait-changing disorders in the area of both lower extremities
3. Follow-up of less than 24 months

Thirty-three patients with corresponding inclusion criteria were invited to undergo gait analysis, Electromyography (EMG) measurement, pedobarography and examination/questioning on a single day for follow-up measurement.

A total of 14 patients (9 men and 5 women) with an average clinical follow-up, on the day of examination, of 80.64 months (SD 36.38, range 37–152) met the inclusion criteria and could be fully examined. The remaining patients of the initial 33 patients could no longer be reached or did not wish to participate.

### Gait analysis

Three-dimensional (3D) biomechanical gait and EMG analyses of the ankle dorsiflexors and plantarflexors muscles were conducted while participants performed five trials of level-ground walking over 10 m at a self-selected pace.

**Fig. 1:**
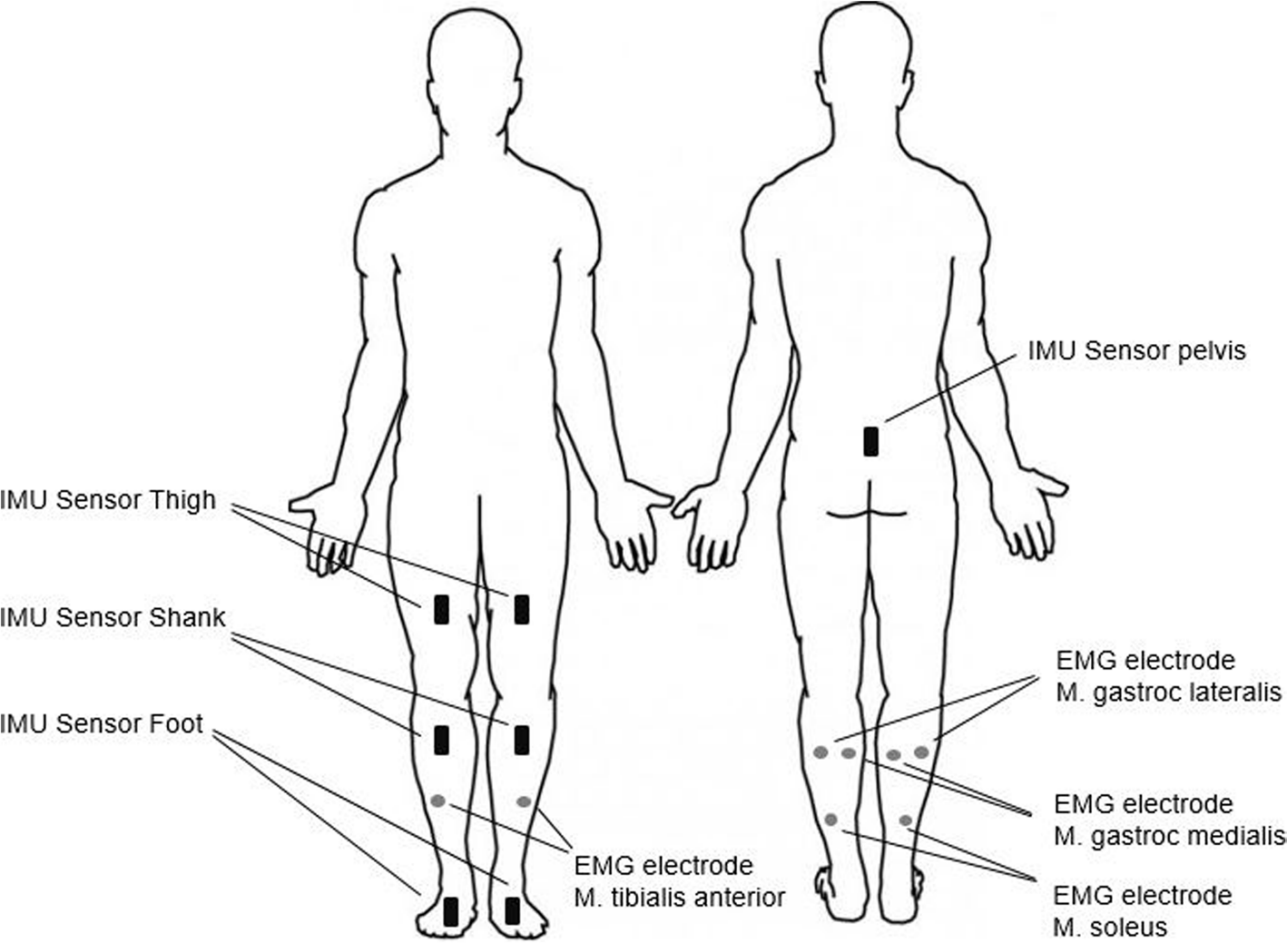
Illustration of the IMU sensors and the EMG electrodes attached to the patient

To that end, the 3D MyoMotion and MyoMuscle (myoRESEARCH 3.18 Software, Noraxon U.S.A. Inc., Scottsdale, Arizona, USA) analysis systems were used as described previously [17, 18]. The 40 × 27 mm large disposable, self-adhesive Ag/AgCL dual snap surface EMG electrodes with an interelectrode distance of 20 mm were positioned on the M. gastroc lateralis, M. gastroc medialis, M. soleus and M. tibialis anterior on both sides (Fig. 1). To reduce the skin resistance of the EMG electrodes, hair on the area where the electrodes were to be attached was removed with a shaver, and the skin was sterilized with alcohol. The sampling rate of the EMG signals was 2000 Hz, and a 10 to 500 Hz bandpass filter and 50 Hz Infinite Impulse Response Notch Filter were used. EMG signals were processed by signal rectification and smoothing with the root mean square (RMS) method with a smoothing window length of 75 ms. The amplitude of the EMG signals recorded during walking was normalized using the submaximal isometric contraction (sMVC) as described for subjects who cannot perform a maximum contraction because of pain, muscle inhibition or risk of injury [19]. Depending on the muscle group, the subjects had to contract sitting or standing against an insurmountable object as strong as they could without pain. The sMVC was set to 100%. The mean EMG in percentage to the sMVC during stance, swing and total were analysed. The inertial measurement unit sensors were calibrated before every trial of walking separately. The system consists of seven sensors who were mounted in designated positions at the pelvis, thighs, shanks and feet to measure the anatomical angles and range of motion (ROM) of the hip, knee and ankle joints. The sampling frequency was 100 Hz. The minimum and maximum peak angles of the joints during whole gait and separated for stance and swing phases of walking were analysed as well as spatial–temporal parameters. Determination of stance and swing phases was based on the acceleration data of the feet sensors and automatically provided by the MyoMotion system. In addition, we performed a pedobarography with a pressure distribution platform (Zebris 1.5 FDM, Zebris Medical GmbH, Isny im Allgäu, Germany) consisting of 11,264 sensors on a 1,440 × 560 mm sensor area (resolution 1.4 sensors / cm^2^) at 100 Hz. For this purpose, the participants walked over a 5-m long walkway where the pressure distribution plate was embedded in (2 m before the plate, 1.5 m plate, 1.5 m after the plate). There were at least three contacts with the ground with alternating foot sides at the start at a self-selected speed in a separate test setup. The data from three trials were averaged for each participant. Force data were normalized to the respective body weight. Some of the force and pressure distribution data is displayed separately in three zones, this corresponds to a preconfigured geometric division of 40% forefoot, 30% midfoot and 30% heel in the software. In addition, a stand analysis was carried out on the pressure distribution plate, in which the test subjects were asked to stand still for 30 s. All gait data were compared between the injured and healthy sides. All patients could walk without aids. The gait analysis was carried out in the patient's shoes, and the pedobarography was performed without shoes.

### Questionnaires

To determine the subjective and functional outcome at least 24 months after Chopart injury, one questionnaire about health-related quality of life (Short Form Health Survey Score—SF-36) and two questionnaires related to functional outcome (American Orthopedic Foot and Ankle Society score—AOFAS Score and the Foot and Ankle Ability Measure Score -FAAM Score-validated German version) were completed [20–22].

Additionally participants were asked to rate their pain on a numeric scale [23].

### Statistical analysis

The results were evaluated after completion of the study using the statistical program (TIBCO Statistica 14.0.0, StatSoft GmbH, Hamburg, Germany). Mean values and standard deviations (SD) were calculated. After test for normality distribution a t test for independent samples was performed for evaluation between the injured and healthy sides with an alpha level of 0.05 as the minimum level of significance.

## Results

### Patients

The average age at the time of the examination was 48.36 (SD 14.73, range 28–72) years. The reported mechanism of trauma is shown in Table 1. The distribution of injuries was as follows: APC 12 (85.71%), navicular 3 (21.42%), cuboid 3 (21.42%) and avulsion fracture talus 3 (21.42%). In no case was there a luxation in the CC (calcaneocuboidal) or TN (talonavicular) joint or a complete luxation.

**Table 1:** Study group

Study group	
**Age at fracture (years)**	40.86 ± 15.98(range 19–67)
**Sex**	
Male	9 (64.29%)
Female	5 (35.71%)
**Surgical therapy**	8 (57.14%)
**Conservative therapy**	6 (42.85%)
**BMI (kg/m2)**	27.66 ± 4.73 (range 20.1–34.6)
**Reason for injury**	
Sprain	7 (50%)
Fall from more than 3 m	2 (14.28%)
Fall less than 3 m	1 (7.14%)
Crush injury	2 (14.28%)
Rollover/traffic accident	2 (14.28%)
**Fractures/avulsion injuries**	
Navicular	3 (21.42%)
Calcaneus	12 (85.71%)
Cuboid	3 (21.42%)
Talus	3 (21.42%)
**Footwear**	
Conventional shoes	8 (57.14%)
Insoles	3 (21.42%)
Orthopaedic made-to-measure shoes	3 (21.42%)
**Comorbidities**	
Smoking	8 (57.14%)
Arterial hypertension	1 (7.14%)
Gastric carcinoma	1 (7.14%)
Lumbar spine fracture	1 (7.14%)

Of the 14 patients, the injury was initially overlooked in four patients, and three of them were subsequently treated surgically (2 × resection APC fragment, 1 × arthrodesis CC joint).

Of the remaining patients, five were treated nonsurgically (tip toe weight bearing for 6 weeks), four were treated surgically (2 × ORIF of the APC, 1 × ORIF APC + naviculare, 1 × arthrodesis CC joint) and one patient required arthrodesis of the CC joint after initial nonsurgical treatment. Further demographic data are shown in Table 1.

### Gait analysis

The differences found for the EMG measurements and kinematics in the whole gait cycle, spatiotemporal parameters, force and pressure parameters during standing measurement and stance phases of gait between the healthy side and the injured side are shown in Tables  2, 3 and 4.

**Table 2:** EMG measurements, kinematic and spatiotemporal parameters

**Parameter**		**Group (mean ± SD)**		**t Test *p* values**
		**healthy**	**injured**	
Gastroc. Lat. (% SMVC)	Mean	34.23 ± 20.88	36.65 ± 22.92	0.773
Gastroc Med. (% SMVC)	Mean	40.72 ± 22.69	49.32 ± 27.08	0.371
Soleus (% SMVC)	Mean	34.37 ± 24.12	31.08 ± 16.53	0.677
Tib. Ant. (% SMVC)	Mean	30.46 ± 22.48	32.66 ± 19.03	0.782
Stance phase (%)		62.48 ± 2.87	62.10 ± 1.72	0.676
Swing phase. (%)		37.52 ± 2.87	37.90 ± 1.72	0.676
Loading response (%)		12.30 ± 1.81	11.85 ± 2.64	0.607
Step length (cm)		70.28 ± 7.09	67.31 ± 7.93	0.306
Step time (ms)		556.96 ± 38.64	564.45 ± 33.27	0.588
	Min	-15,15 ± 4,37	-14,33 ± 4,01	0,610
Hip Flexion (°)	Max	33,49 ± 5,73	32,50 ± 5,77	0,651
	ROM	48,64 ± 5,63	46,83 ± 6,92	0,454
	Min	-9,49 ± 3,03	-9,90 ± 3,39	0,742
Hip Abduction (°)	Max	7,96 ± 4,08	5,56 ± 3,40	0,103
	ROM	17,45 ± 4,87	15,45 ± 4,30	0,261
	Min	-5,41 ± 3,43	-7,96 ± 2,42	0,032*
Hip Rotation (°)	Max	10,01 ± 5,27	6,30 ± 4,17	0,049*
	ROM	15,42 ± 4,32	14,26 ± 4,65	0,500
	Min	-13,71 ± 5,06	-12,65 ± 6,78	0,644
Ankle Dorsiflexion (°)	Max	13,80 ± 6,09	13,54 ± 6,63	0,913
	ROM	27,51 ± 6,52	26,18 ± 6,86	0,605
	Min	-9,06 ± 3,74	-10,76 ± 6,56	0,409
Ankle Inversion (°)	Max	19,45 ± 7,92	12,51 ± 14,11	0,121
	ROM	28,51 ± 9,33	23,27 ± 14,57	0,267
	Min	-13,94 ± 6,98	-8,33 ± 5,89	0,030*
Ankle Abduction (°)	Max	7,18 ± 4,51	7,38 ± 2,30	0,882
	ROM	21,12 ± 6,23	15,71 ± 6,29	0,031*
	Min	-0,79 ± 5,08	-0,34 ± 4,21	0,800
Knee Flexion (°)	Max	64,43 ± 5,99	62,14 ± 7,87	0,394
	ROM	65,22 ± 5,66	62,47 ± 7,53	0,286
	Min	-5,58 ± 2,83	-6,33 ± 2,55	0,464
Knee Rotation (°)	Max	10,13 ± 4,95	9,91 ± 3,96	0,898
	ROM	15,71 ± 4,67	16,25 ± 3,56	0,735
	Min	-5,98 ± 4,47	-3,96 ± 3,99	0,218
Knee Abduction (°)	Max	6,76 ± 4,16	8,54 ± 7,35	0,436
	ROM	12,74 ± 4,46	12,50 ± 5,81	0,906

^*^ statistically significant. All EMG and kinematic values are for the whole gait cycle

Hip Flexion: positive values are flexion, negative values are extension; Hip Abduction: positive values are abduction, negative values are adduction; Hip rotation: positive values are external rotation, negative values are internal rotation

Knee Flexion: positive values are flexion, negative values are extension; Knee Abduction: positive values are abduction, negative values are adduction; Knee rotation: positive values are external rotation, negative values are internal rotation

Ankle Dorsiflexion: positive values are dorsiflexion, negative values plantarflexion; Ankle Abduction: positive values are abduction, negative values are adduction; Ankle Inversion: positive values are inversion, negative values are eversion

**Table 3:** Force and pressure parameters gait analysis

**Parameter**		**Group (mean ± SD)**		**t Test *p *values**
		**healthy**	**injured**	
Mean Force (N/kg)	Forefoot	1.11 ± 0.04	1.04 ± 0.19	0.022*
Mean Force (N/kg)	Rearfoot	0.76 ± 0.07	0.76 ± 0.07	0.734
Max. Force 1 (N/kg)	Whole foot	1.10 ± 0.05	1.08 ± 0.06	0.150
Max. Force 2 (N/kg)	Whole foot	1.13 ± 0.04	1.11 ± 0.06	0.016*
**3 Zones analysis**				
Max Force (N/kg)	Forefoot	1.05 ± 0.05	1.00 ± 0.20	0.097
	Midfoot	0.25 ± 0.12	0.27 ± 0.13	0.593
	Heel	0.67 ± 0.09	0.65 ± 0.10	0.371
Max Pressure (N/cm^2^)	Forefoot	45.41 ± 14.33	44.36 ± 19.51	0.779
	Midfoot	17.92 ± 9.06	14.43 ± 4.85	0.030*
	Heel	29.32 ± 6.05	29.03 ± 6.21	0.824

**Table 4:** Force and pressure parameters stance analysis

**Parameter**		**Group (mean ± SD)**		**t Test *p* values**
		**healthy**	**injured**	
Load distribution (%)	Forefoot	47.90 ± 9.04	43.57 ± 12.29	0.298
	Heel	52.11 ± 9.04	56.43 ± 12.29	0.299
	Total	52.85 ± 5.10	47.15 ± 5.10	0.007*

**Fig. 2:**
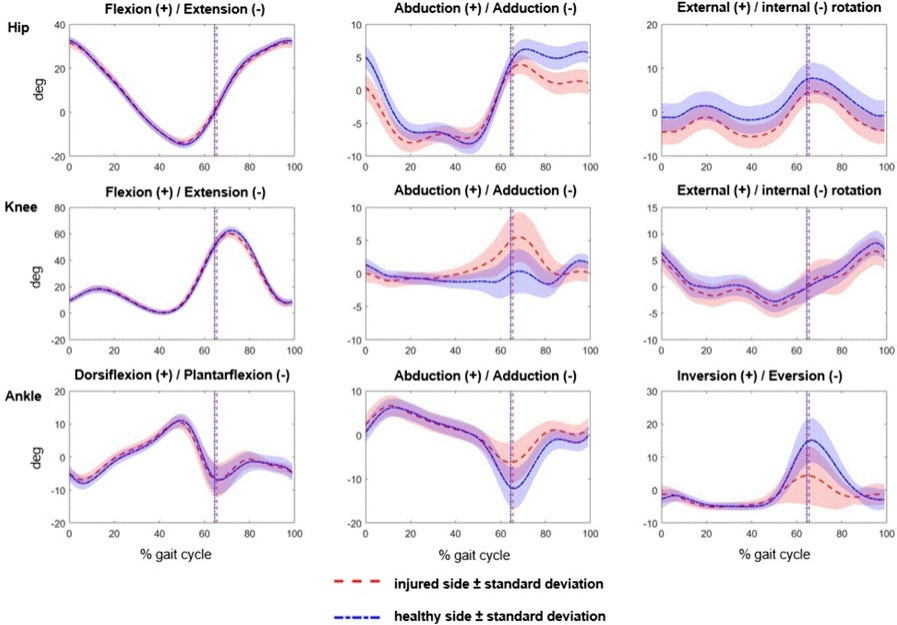
Kinematic curves of the injured side (red) in relation to the healthy side (blue) shown as time normalized averaged curves of all participants plus/minus 1 standard deviation. The direction of movement listed first in the heading always corresponds to the positive values. In the case of rotation, positive values are external rotation and negative values are internal rotation

Averaged kinematic curves of the injured side in relation to the healthy side are demonstrated in Fig. 2.

The following significant differences were found: During hip rotation in the whole gait cycle, there was increased internal rotation (*p* = 0.032) and decreased external rotation (*p* = 0.049) on the injured side compared to the uninjured side. However, the ROM was not significantly changed (*p* = 0.500).

In addition, there was significantly reduced adduction on the injured side (*p* = 0.030) and reduced ROM on the injured side (*p* = 0.031) in the ankle area during whole gait cycle. Furthermore, a significantly increased mean force in the forefoot (*p* = 0.022) and an increased second maximum force peak were found on the healthy side compared to the injured side during stance phase of gait (*p* = 0.016). Additionally, the maximum pressure in the midfoot on the healthy side was higher than that on the injured side (*p* = 0.030).

During stand measurement, the healthy side was subjected to a significantly higher load compared to the injured side (*p* = 0.007).

No significant differences were found in the percentage distribution of the gait phases, spatiotemporal parameters or EMG measurements. Table 2 shows only the EMG data for the entire gait cycle, as the separate data for stance and swing phase did not yield any results worth mentioning.

### Questionnaires

The subjective and functional outcome scores are shown in Tables 5 and 6. The mean FAAM-ADL and FAAM-Sport scores were 82.84 (SD 18.87, range 39–100) and 77.40 (SD 28.02, range 15–100), respectively, out of a maximum of 100%. The mean AOFAS score was 83.29 (SD 20.4, range 29–100) out of a maximum of 100 points. The mean score on the numerical rating scale for pain was 1.36 (SD 1.95, range 0–6) out of a maximum of 10 points. The SF-36 score showed a physical component summary of 46.41 (SD 8.84, range 29.47–59.23) and a mental component summary of 49.92 (SD 11.88, range 24.2–59.49).

**Table 5:** Results of the FAAM Score, AOFAS Score and Numeric Rating Scale for pain

	**FAAM-ADL (%)**	**FAAM -ADL global (%)**	**FAAM Sport (%)**	**FAAM—Sport global (%)**	**FAAM overall level**	**AOFAS** **(max 100 points)**	**Pain** **1 = no pain** **10 = max pain**
1	88.1	85	85	80	almost normal	97	0
2	100	100	92.9	90	normal	100	0
3	96.4	90	100	90	almost normal	90	0
4	52.5	50	87.5	50	abnormal	72	3
5	96.4	90	100	90	normal	98	0
6	66.6	75	32.1	50	abnormal	77	2
7	100	100	100	100	normal	100	0
8	76.2	70	67.9	70	almost normal	72	1
9	88.8	85	71.4	80	almost normal	86	3
10	38.7	40	15	0	abnormal	29	6
11	100	90	100	80	almost normal	100	0
12	77.4	90	43.8	60	almost normal	58	4
13	92.9	100	96.4	50	almost normal	90	0
14	85.7	90	91.6	70	almost normal	97	0

**Table 6:** SF- 36 scores

SF 36	Physical functioning	Role limitations (physical health)	Role limitations (emotional problems)	Energy/ fatigue	Emotional well-being	Social functioning	Pain	General health	Physical Component Summary	Mental Component Summary
Mean	76.07	69.64	76.19	55.71	74	83.04	60.57	63.43	46.41	49.92
SD	21.05	46.18	40.15	18.38	18.41	24.81	27.08	21.56	8.84	11.88
Minimum	25	0	0	10	28	25	22	15	29.47	24.2
Maximum	100	100	100	80	88	100	100	97	59.23	59.49

## Discussion

To our knowledge, this is the first study on gait analysis after isolated Chopart injury. Biomechanical analysis and particularly pedobarography have been widely demonstrated to be a relevant measure of lower extremity function following trauma or degenerative pathology of the foot [18, 24–28]. The strength of our study is that all 14 patients completed a full gait analysis, EMG measurement and pedobarography and provided outcome scores so that an overall picture of the changes resulting from this injury emerged. The therapy procedure in this group of patients varied, but there were too few patients to make a differentiation. Nevertheless, we wanted to determine whether and in what way there was a change in the gait pattern. We were able to show that isolated injuries in the area of the Chopart joint led to significant changes in gait and kinetic data. The increased internal rotation in the hip and the reduced ROM of the ankle with reduced adduction could suggest a redistribution of load to the lateral column in the injured foot, as described by Kinner et al. [29] in a pedobarography of plantar pressure in calcaneus fractures involving the CC joint. Our data showed increased maximum force values in the injured foot for the midfoot; however, these remained nonsignificant, and discrimination of foot columns was not possible in our set up. The reduced adduction, in turn, could be due to the injury of the Chopart joint since this is proportionally involved in the adduction of the foot in the uninjured state [16]. This could also explain the increased internal rotation of the hip in terms of compensation. Another study by Richter et al. [30] described abnormal to severely abnormal results after pedobarography in five patients after Chopart fracture dislocation without going into further detail. Other changes in gait were not examined in either study. We were also able to show that the uninjured side is loaded more than the injured side during standing and that the mean vertical ground reaction force in the forefoot as well as the maximum pressure in the midfoot during walking are significantly lower on the injured side compared to the uninjured side. A study by Kösters et al. [31] showed a reduced force–time integral in the injured foot, which is in line with our results; this suggests that in the stance phase, there is less weight on the injured foot than on the healthy side. This is probably explained by the fact that the highest load on the Chopart joint is reached during push-off, and patients try to reduce this load [16]. However, injuries to the Chopart joint do not seem to have a major impact on muscle activity, especially the triceps surae complex (M. soleus, M. gastrocnemius med. and lat.), although these affect the Chopart joint and are essentially responsible for the powerful push-off. In our study, no significant difference between the healthy side and injured side was found. Klos et al. [16] equally concluded that passive structures appear to be more important than muscles in stabilizing the foot in the Chopart joint during the stance phase.

Animal experiments and epidemiological studies have shown that changed stress on joints and abnormal stress or normal stress in an abnormal direction are key factors for the development of osteoarthritis, so the long-term consequence can be expected to be premature osteoarthritis not only in the joints of the lateral foot column but also in other joints of the lower extremities [32].

A recent study by Rammelt and Missbach [4] showed that overall, functional restrictions could be determined in the long-term outcome after injuries in the Chopart joint. The PSC and MSC of the SF-36 were 46.4 and 53.3 in 33 patients with isolated Chopart injury, thus confirming the values of 46.41 and 49.92 that we recorded. The average AOFAS score was 78.2 (*n* = 33) with isolated injuries of the Chopart joint. Our study also identified a limitation with an average value of 83.3. Studies by Van Dorp et al. [2] and Richter et al. [30] showed even poorer results, averaging 72 and 75 points, respectively, but both studies included patients with injuries outside the Chopart series. The values of the FAAM score collected in this study also confirm limitations in activities of everyday life and even more limitations in sporting activities after isolated Chopart injury. Although the patients examined in this study did not have any dislocations in the CC or TN joint, fractures that initially appear simple, such as APC fractures, can lead to changes and should not be underestimated. There may also be ligamentous injuries, which can usually be diagnosed only with an additional MRI examination [14], which was not performed in our study.

### Limitations

The number of patients examined here is small. However, this is due on the one hand to the low incidence of this injury and on the other hand to the rate of concomitant fractures of 75% to 90%, which was an exclusion criterion for this study. In addition, the patient population is inhomogeneous since it also includes patients with arthrodesis. However, since this is often the consequence of a Chopart injury, these patients were included. Only bony injuries were listed in this study. No statement can be made about possible ligamentous injuries due to the lack of diagnostics such as MRI. The gait analysis was carried out with shoes in order to be able to understand the gait in everyday life, at work and over longer distances. Pedobarography, on the other hand, was measured without shoes. The measurements were not compared with a healthy control group without lower extremity pathology. It is therefore not possible to say whether the injury has an impact on the healthy side as found in other studies [33, 34].

By using the manufacturer's software when analysing pedobarography, a reduction to three foot zones was made without separating the toes and differentiating between the medial and lateral foot column, which limited the comparability with other studies. Due to the injury situation of our patients, no maximal voluntary contraction could be performed before the EMG measurements and the sMVC was used. This limits comparisons between muscles or individuals.

## Conclusions

Isolated injuries of the Chopart series not only affect global foot function but also affect the overall gait pattern and range of motion of other joints of the lower extremities. In addition, there are functional and subjective impairments over the long term.

## Data Availability

The datasets used and/or analysed during the current study available from the corresponding author on reasonable request.

## Abbreviations

APC: Anterior process of the cacaneus
AOFAS Score: American Orthopedic Foot and Ankle Society score
BMI: Body-Mass-Index
CC: Calcaneocuboidal
EMG: Electromyography
FAAM Score: The Foot and Ankle Ability Measure Score
ROM: Range of motion
RMS: Root mean square
SF-3: Short Form Health Survey Score
sMVC: Submaximal isometric contraction
TN: Talonavicular
